# Genome-Wide Association Study Suggests *rrp44* Is a Key Regulator of Growth Traits in Channel Catfish (*Ictalurus punctatus*)

**DOI:** 10.3390/cimb48040420

**Published:** 2026-04-18

**Authors:** Shiyong Zhang, Hongyan Liu, Yongqiang Duan, Minghua Wang, Xiaohui Chen

**Affiliations:** National Genetic Breeding Center of Channel Catfish, Freshwater Fisheries Research Institute of Jiangsu Province, Nanjing 210017, China; shiyongzhang@hotmail.com (S.Z.); njsfdxliuhongyan@163.com (H.L.); yqduan0712@163.com (Y.D.); minghua.wang@aliyun.com (M.W.)

**Keywords:** channel catfish, whole-genome resequencing, genome-wide association study (GWAS), growth traits, *rrp44*

## Abstract

Understanding the genetic architecture underlying growth variation is central to improving aquaculture species through genomic selection. Here, we performed a genome-wide association study (GWAS) on 303 individuals from a G_2_ breeding population of channel catfish (*Ictalurus punctatus*) using whole-genome resequencing data. After stringent quality control, 5.64 million high-confidence single nucleotide polymorphisms (SNPs) were retained for association analyses of two key growth traits—monthly weight gain (MWG) and body depth (BH). We identified 15 and 28 loci significantly associated with MWG and BH, respectively, with the majority concentrated on chromosome 20. Two SNPs (Chr20:14,657,971 and Chr20:14,658,012) located in exon 9 of the *rrp44* gene were significantly associated with both traits. Functional annotation and enrichment analyses revealed that the *rrp44* gene, encoding an exoribonuclease subunit of the RNA exosome complex, participates in mitotic spindle regulation and post-transcriptional RNA decay, processes critical for cellular growth and metabolic homeostasis. We propose that *rrp44* may influence growth through the modulation of feeding rhythm and circadian regulation, providing a potential molecular basis for growth heterogeneity in channel catfish. These findings enrich our understanding of growth-related genomic variation and offer valuable molecular markers for precision breeding and genetic improvement of catfish.

## 1. Introduction

Genome-wide association analysis (GWAS) aims to utilize single nucleotide polymorphisms (SNPs) across the entire genome to explore the relationship between genetic and phenotypic variations. This research approach was initially proposed by Risch and Merikangas [1], marking a pivotal shift in the study of complex human diseases from focusing on candidate genes to a genome-wide perspective. In 2005, Klein et al. pioneered the application of GWAS methodology by investigating the association between 116,204 SNPs in the human genome and age-related macular degeneration (AMD), successfully pinpointing the *CFH* gene as a significant candidate gene for this condition [2]. Subsequently, GWAS has facilitated the discovery of numerous genetic variations and candidate genes associated with various traits and complex genetic disorders.

The development of genetic research approaches has progressed from early Sanger sequencing and PCR-based marker methods (e.g., RFLP, SSR) to next-generation sequencing (NGS) technologies. This transition has dramatically increased throughput while reducing costs, enabling genome-wide genotyping at an unprecedented scale. Consequently, GWAS has progressively found applications in genetic analyses across diverse traits in both animals and plants. Its utilization in breeding initially gained traction in the domain of dairy cows. Daetwyler et al. [3] and Pryce et al. [4] sequentially identified several genes within Quantitative Trait Loci (QTL) associated with milk production and fertility traits in dairy cows. In the realm of aquatic animal genomics, GWAS has emerged as a prominent research tool in recent years, offering substantial potential for deepening our understanding of the genetic underpinnings regulating the manifestation of complex quantitative traits and for identifying potential functional genes and molecular markers.

To date, numerous GWAS studies based on aquatic animal populations have been documented, encompassing a wide array of traits such as growth [5,6,7], reproduction [8], meat quality [9,10], extreme temperature tolerance [11], low oxygen tolerance [12], saline tolerance [13], ammonia tolerance [14], and disease resistance [15]. For instance, Sodeland et al. [9] pioneered the use of Illumina Iselect SNP array to conduct whole-gene association analyses on fat content and filet texture traits in Atlantic salmon, ultimately mapping fat content QTL on chromosomes 9 and 10, and fillet firmness QTL on chromosomes 3 and 11. Similarly, Wang et al. [12] employed a 250 K SNP array of channel catfish to identify SNPs associated with hypoxia tolerance from a cohort of 376 channel catfish representing six strains, uncovering a genomic region on LG6 significantly linked to hypoxia tolerance traits. Additionally, Zhou et al. [15] employed a similar approach to screen SNPs and genes associated with enteric septicemia in 499 channel catfish, confirming a significant association between disease resistance and a genomic region on LG1, with 14 genes in this region exhibiting immunological functions. Thus, GWAS has emerged as a pivotal approach for dissecting the genetic basis of various aquatic animal traits, as evidenced in species such as large yellow croaker [16], grouper [5,7], rainbow trout [6,10], and others.

The growth trait stands out as one of the most economically significant characteristics in fish breeding, representing a multifaceted physiological process influenced by numerous factors including genetic and environmental elements [17], feeding practices [18], and feed composition [19]. Numerous studies have underscored the moderate heritability of growth [20,21,22,23], indicating that continuous selection of genetic variations can notably enhance offspring growth performance through marker-assisted selection. QTL mapping and GWAS stand out as two pivotal methods for identifying markers and genes linked to growth traits. Both genotyping techniques, including high-density arrays and NGS, offer distinct strategies for developing tens of millions of genome-wide molecular markers for GWAS. These approaches facilitate the elucidation of the genetic basis underlying growth traits, contributing to the refinement of breeding programs aimed at enhancing growth performance in fish populations.

Channel catfish (*Ictalurus punctatus*) holds significant importance in aquaculture globally. Since its introduction to China in the 1980s, the yield of this species has seen a remarkable increase, stabilizing at approximately 5 × 10^9^ kg in recent years [24]. Consequently, China has emerged as a key producer and marketer of catfish. In this study, we employed the BGISEQ-500 platform to resequence the genome of channel catfish from different families with similar initial specifications. Subsequently, we conducted an analysis to explore the association between SNP variations and two growth traits: monthly weight gain and body depth. Our objective was to identify pivotal candidate genes and molecular markers associated with growth. To mitigate the influence of environmental factors on experimental outcomes, all offspring from the families were reared under uniform environmental conditions. The findings of our study offer valuable insights into the molecular mechanisms regulating growth traits in channel catfish. Moreover, the significant molecular markers identified could serve as valuable resources for subsequent genomic selection breeding programs aimed at enhancing growth performance in channel catfish.

## 2. Materials and Methods

### 2.1. Ethics Statement

All experiments were performed in accordance with the Regulations of the Animal Ethics Committee and were approved by the Institutional Review Board on Bioethics and Biosafety of Freshwater Fisheries Research Institute of Jiangsu Province (No. FT 20196).

### 2.2. Samples and Phenotype Data

The samples used in this study were obtained from the G_2_ generation breeding population, which was bred at the National Genetics & Breeding Center of Channel Catfish located in Nanjing, Jiangsu Province, China. In May 2009, five populations of channel catfish imported from the United States were utilized as foundational breeding populations to establish G0 generation breeding lines. These populations included Texas (1997), Arkansas (1999), Mississippi (2001), Arkansas (2003), and Arkansas (2004). Subsequent breeding populations were systematically developed across generations. Breeding values were computed based on growth data and pedigree information. In June 2017, a total of 49 full-sib G_2_ generation families were generated from 30 sires and 49 dams originating from the parental catfish (G_1_).

The breeding methodology has been previously documented [25]. Approximately 100 juveniles of similar size were randomly selected from each family at 180 days after hatching (dah), and their growth parameters, including body weight and body length, were meticulously recorded. Each fish was implanted with a PIT tag (1.4 mm × 8 mm) containing a 12-digit identification code (Haixing Instrument Co., Ltd., Qingdao, China) into the abdominal cavity through the pelvic fin. Once the wounds had completely healed, all experimental catfish were transferred to a 0.2 hm^2^ pond for cultivation. During the rearing process, a feeding regimen based on the principle of “fixed time, fixed position, and fixed quantity” was strictly adhered to. At about 540 dah, the experimental fish were retrieved, and their growth parameters, including body weight and body depth, were once again recorded. Sex was initially determined by visual examination of gonadal morphology at the time of the second phenotypic measurement and subsequently confirmed using sex-linked molecular markers [26]. Monthly weight gain (MWG) was calculated as (final body weight − initial body weight)/number of months, where the number of months corresponds to the time interval between the first and second body weight measurements. Body depth was measured at the second time point, calculated as the vertical distance from the dorsal fin origin to the ventral margin. During the second measurement of body weight and phenotypic data, caudal fin samples were collected from each catfish and preserved at −80 °C for further analysis.

### 2.3. Library Construction and Sequencing

A total of 303 catfish samples were randomly selected from the collected experimental specimens. Genomic DNA from each sample was extracted and purified using the Animal Tissue DNA Extraction Kit (Vazyme, Nanjing, China) following the manufacturer’s instructions. Subsequently, sample quality was assessed using a Qubit Fluorometer (ThermoFisher, Waltham, MA, USA) and 1% agarose gel electrophoresis. Only DNA samples meeting the following criteria were deemed suitable for further analysis: total content > 3 µg; concentration > 30 ng/µL; OD260/OD280 = 1.8~2.0.

The genomic DNA of each sample was then fragmented using the Covaris E220 focused ultrasonicator (Covaris, Brighton, UK) into fragments ranging from 50 to 800 bp. Fragments ranging from 100 to 300 bp were selectively isolated using the Agencourt AMPure XP magnetic bead-based purification system (Beckman, Krefeld, Germany). To create sticky-end DNA, the ends of the isolated DNA fragments were repaired, and dATP was added to the 3′ end. Unique barcode sequences with dTTP tails were ligated to both ends of each DNA fragment to enable differentiation between catfish samples. Subsequently, single-stranded circular DNA was generated through rolling circle amplification (RCA) via 8 cycles of amplification.

Single-stranded circularization was achieved through the following steps: denaturation of the PCR product with a specific molecule, the inverse complementary molecule to a specific strand of the PCR product, followed by ligation of the single-stranded molecule with DNA ligase. Any remaining linear molecules were digested by exonuclease to yield single-stranded circular DNA. Each library contained 16 samples, except for the final library, which contained 15 samples. Libraries were allocated according to sample numbers that had been randomly assigned at the time of sampling. Finally, the 303 samples were grouped to construct 19 sequencing libraries, all of which were sequenced on the BGISEQ-500 platform (BGI-Qingdao, Qingdao, China) with 100 bp paired-end reads.

### 2.4. Genotyping and Imputation

Bioinformatics analysis commenced upon receipt of the raw data generated by the BGISEQ-500 sequencing platform. To enhance sequence quality, Trimomatic software (version 0.36, http://www.usadellab.org/cms/index.php?page=trimmomatic (accessed on 9 May 2025)) was employed to filter the raw data. The forward and reverse fastq sequences of each sample served as input files, with software parameters set as “LEADING:3 TRAILING:3 SLIDINGWINDOW:4:15 MINLEN:75”.

The Picard MarkDuplicates tool (version 2.26.1, https://broadinstitute.github.io/picard/ (accessed on 9 May 2025)) was then utilized to eliminate PCR duplicates. Subsequently, the high-quality clean reads were aligned to the reference genome sequence of channel catfish [27] using the default parameters of BWA software (version 0.7.17, http://bio-bwa.sourceforge.net/ (accessed on 9 May 2025)). SAM format files were imported into SAMtools software (version 1.16.1, http://samtools.sourceforge.net/ (accessed on 11 May 2025)) [28] for sorting and indexing the uniquely mapped reads. The unified genotype module of GATK software (version 2.4-9, http://www.broadinstitute.org/gatk/ (accessed on 13 May 2025)) [29] was then employed to call SNPs from valid BAM files. To mitigate SNP errors stemming from incorrect alignment, adjacent SNPs were required to be at least 5 bp apart. Subsequently, all SNPs from the 303 samples underwent further filtering using VCFtools (version 0.1.10) [30], following the filtering parameters described previously [14]: (1) minimum and maximum allelic number = 2; (2) minor allele frequency (MAF) > 0.05; and (3) rate of missing data < 0.05. Missing SNPs were imputed using Beagle v5.0 software [31].

Additionally, Vcftools v. 0.1.16 software was employed to determine the genetic locations of all variations. Subsequently, captured variations were annotated using ANNOVAR software (v2018Apr16, http://www.openbioinformatics.org/annovar/ (accessed on 13 May 2025)) [32] according to the gene annotation file of the reference genome.

### 2.5. Assessment of the Genetic Relatedness

To provide a more precise visualization of the population relatedness, a heatmap of kinship values was generated using the GAPIT v3.6.0 software [33].

### 2.6. Genome-Wide Association Study

GWAS was conducted utilizing efficient mixed-model association expedited (EMMAX) [34] within the SNP and Variation Suite v8.x (Golden Helix, Inc., Bozeman, MT, USA, www.goldenhelix.com (accessed on 23 May 2025)), which operates on the basis of a mixed linear model (MLM). The EMMAX statistical test method was chosen due to its superior ability in accounting for sample structure compared to principal component analysis. In this investigation, EMMAX was employed to rectify confounding effects arising from subgroup structure and individual correlations. In this study, the EMMAX mixed linear model was used for GWAS analysis as follows:

**y** = 1***μ*** + **x***_k_β_k_* + **C*****γ*** + **g** + ***ε***

where **y** is the phenotype vector for monthly weight gain (MWG) or body depth (BH); ***μ*** is the overall mean; **x***_k_* is the genotype vector for the *k*-th SNP (coded as 0, 1, or 2); *β_k_* is the additive effect of that SNP (to be tested); **C** is the covariate matrix, including: sex (male/female, as a fixed effect), the first five principal components (calculated from genome-wide SNPs to control population structure), and family effect (treated as either a random or fixed effect, depending on the model); **γ** is the vector of regression coefficients for the covariates; **g** is the polygenic random effect, **g**∼*N*(0, σ^2^*_g_**K***); ***ε*** is the residual error, ***ε***∼*N*(0, σ^2^*_e_**I***); ***K*** is the kinship matrix calculated from all SNPs (using GAPIT). The variance components (σ^2^*_g_* and σ^2^*_e_*) were estimated once using the REML method, followed by association tests for all SNPs. A stringent threshold for significant associations between all SNPs and the target trait was set at *p* ≤ 1 × 10^−6^ (equivalent to −log10(*p*) ≥ 6).

## 3. Results

### 3.1. Evaluation of Growth-Related Traits

A total of 303 catfish samples were randomly selected from 49 full-sib G_2_ families with a rearing cycle of approximately 12 months, and each family contributed five to nine individuals. Among the 303 individuals, 156 were males and 147 were females. Growth data, including body weight, body length, and body depth, were collected before and after the rearing period. Prior to stocking, each catfish was injected with a PIT electronic marker to record its identity information. The PIT tag retention rate over the 18-month experimental period was approximately 97.5%, based on routine scanning records.

**Figure 1:**
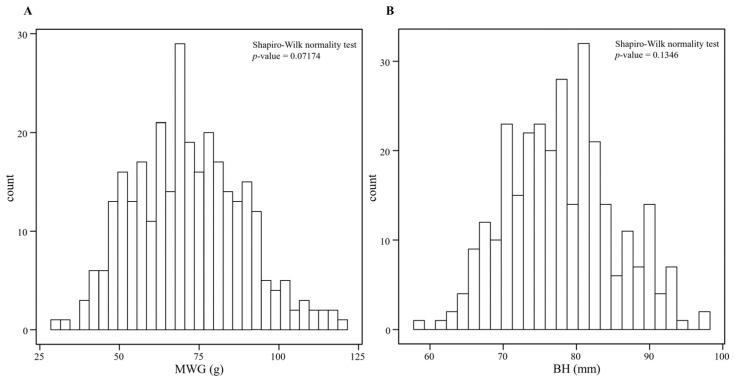
Frequency distribution of MWG (**A**) and BH (**B**) traits of channel catfish.

For the genome-wide association analysis of growth traits, the collected data included monthly weight gain (MWG; in grams) and body depth (BH; in millimeters). The average MWG was 71.73 (±17.16) g, ranging from 29.16 to 119.40 g, while the average BH was 77.95 (±7.34) mm, ranging from 58.13 to 97.85 mm. The normal distribution of the data was assessed using the Shapiro–Wilk test. The results indicated that the significant levels of MWG (Figure 1A) and BH (Figure 1B) were 0.07174 and 0.1346, respectively, both exceeding 0.05. This confirms that the growth traits utilized in this study follow a normal distribution and are suitable for subsequent GWAS research.

### 3.2. Filtering of Raw Data and Calling of SNPs

**Figure 2:**
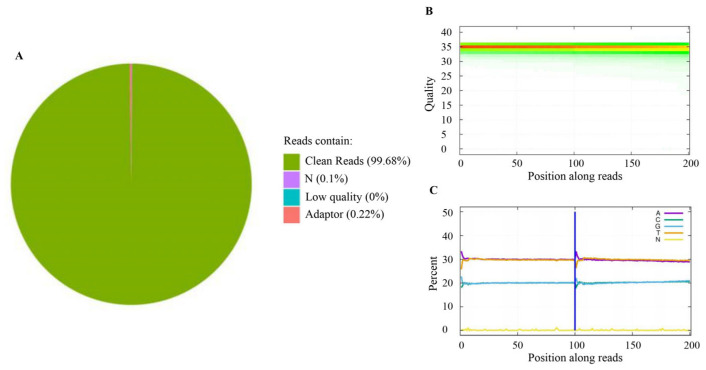
Quality assessment of resequencing data of channel catfish genome. (**A**) Classification of raw reads; (**B**) distribution of qualities, the abscissa represents the position of the base in the read, the ordinate represents the quality value of the base, and each point in the graph represents the total number of bases whose position reached a certain quality value; (**C**) base percentage composition along reads, the abscissa represents the position on the reads, and the ordinate represents the percentage of bases.

In the present study, the genomes of 303 catfish were resequenced using the BGISEQ-500 platform, generating a total of 3.69 TB of raw data, with an average of 12.18 GB per sample. After the removal of barcodes and low-quality sequences, 3.64 TB of clean data was retained, averaging 11.91 GB per sample (ranging from 6.8 to 26.20 GB per sample), with average Q20 and Q30 values of 97.81% and 93.05%, respectively. The mean coverage depth was 14.1× (ranging from 8× to 31×). The average mapping rate was 94.85%, ranging from 90.05% to 96.99%. The classification of raw reads, base mass, and content distribution of clean reads are illustrated in Figure 2.

A total of 12,045,859 original SNPs were called by aligning the clean reads of each sample with the reference genome sequence [27]. Subsequently, after filtering by VCFtools, 5,641,711 SNPs were identified. The number of SNPs on the 29 chromosomes ranged from 55,825 (Chr16) to 309,930 (Chr13), with an average of 194,541 SNPs per chromosome. The density of SNPs ranged from 3.94 to 9.53 SNPs per kilobase (KB), with an average density of 8.01 SNPs per KB (Table 1).

### 3.3. Genetic Relatedness Analysis

**Figure 3:**
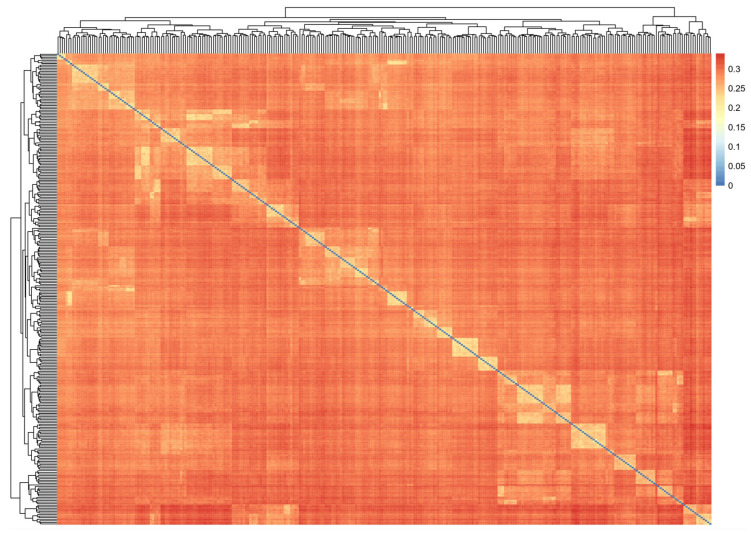
Heatmap of genetic kinships among the 303 samples.

To explore the genetic relationship among the 303 samples in this study, 5,641,711 SNPs from the whole genome were selected. The efficient mixed-model association (EMMA) algorithm was then employed for genetic correlation analysis, as depicted in Figure 3. The results indicated that the genetic correlation among the samples ranged from 0.2 to 0.3, suggesting a robust genetic correlation among them. Furthermore, as the samples utilized in this study have been selectively bred for three generations, the results of the correlation analysis further confirmed that all samples originated from the selected population.

### 3.4. Genome-Wide Association Study

**Figure 4:**
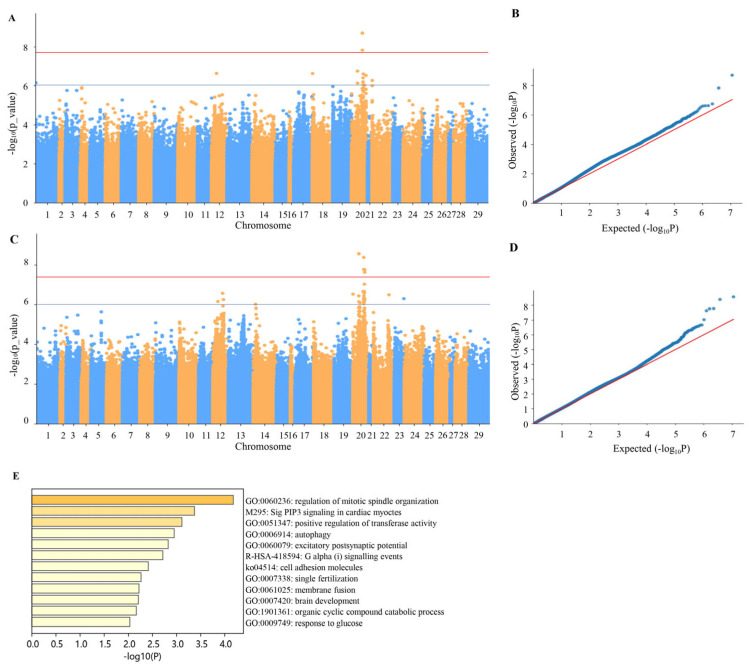
Manhattan and quantile–quantile (QQ) plots of the GWAS data created by EMMAX statistical method. Manhattan plot (**A**) and QQ plot (**B**) of monthly weight gain (MWG) trait; Manhattan plot (**C**) and QQ plot (**D**) of body depth (BH) trait; (**E**) GO enrichment analysis. The gray lines indicate the threshold (−log10(1 × 10^−6^) = 6) for genome-wide significance.

Quantile–quantile (QQ) plots and Manhattan plots were constructed based on the 5,641,711 SNPs (Figure 4). Fifteen markers exhibited significant correlations with monthly weight gain (MWG) (*p* ≤ 1 × 10^−6^, −log10(*p*) ≥ 6) (Figure 4A), while 28 markers were significantly associated with body depth (BH) (*p* ≤ 1 × 10^−6^, −log10(*p*) ≥ 6) (Figure 4B). Notably, over 70% of the significant loci were situated on chromosome 20, with a smaller number of loci detected on chromosomes 12, 14, 18, 22, and 23. Five significant loci were found to be correlated with both traits, as detailed in Table 2. The QQ plots provided confirmation of the credibility and reasonableness of these results (Figure 4C,D).

Furthermore, employing −log10(*p*) = 6 as a threshold, genes within 100 KB upstream and downstream of significant associated SNPs were scrutinized with GO enrichment analysis (Figure 4E). Among these pathways, the mitotic spindle regulatory pathway emerged as the most prominent, with functions primarily related to cell proliferation, tissue growth, and organic matter digestion. This finding further supports the notion that the gene proximal to the SNP site may play a role in regulating the growth of channel catfish, serving as a crucial growth-related quantitative trait locus.

**Figure 5:**
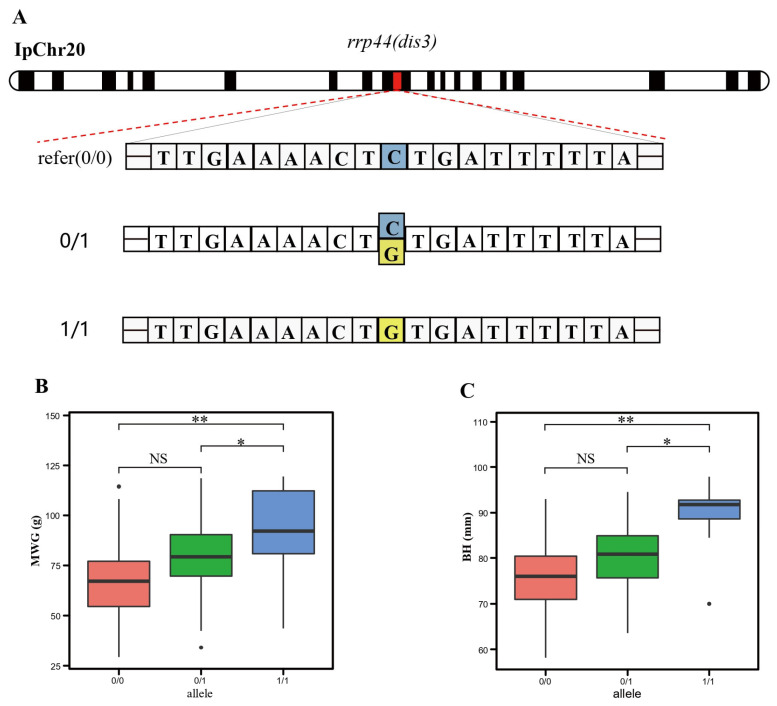
Variation pattern of SNP Chr20-14 658 012 (**A**) and effect of genotype on monthly weight gain (MWG) (**B**) and body depth (BH) (**C**). *, *p* < 0.05; **, *p* < 0.01.

Among these loci, SNP sites 14,657,971 and 14,658,012 on chromosome 20, both located within exon 9 of the *rrp44* gene, exhibited exceptional significance with both traits. These two SNPs were widely distributed across the 49 families, with minor allele frequencies (MAF) of 0.1997 and 0.2013, respectively, across the entire population. This suggests that the SNPs are not lineage-specific but rather represent common variants segregating throughout the breeding population. Specifically, SNP site 14658012 featured a pyrimidine-to-purine mutation (C-G) (Figure 5A), with individuals possessing the GG genotype displaying higher monthly weight gain (MWG) (Figure 5B) and body depth (BH) (Figure 5C) compared to individuals with the other two genotypes (Tukey’s HSD, *p* < 0.05). The effect size (β) was 5.82 g per copy of the G allele for MWG (SE = 1.21, *p* = 1.97 × 10^−9^), and this SNP explained 8.4% of the phenotypic variance in MWG. For BH, the effect size was 2.45 mm per copy of the G allele (SE = 0.52, *p* = 4.11 × 10^−9^), explaining 6.9% of the phenotypic variance.

## 4. Discussion

Channel catfish is an excellent model for genetic studies of phenotypic evolution of fish. During the forty years of its introduction to China, an array of distinctive local breeds was developed and applied. These breeds were genetically adapted to new aquaculture environments, such as Jiangfeng No.1, known for their high yield. The most fundamental purpose of catfish genetic breeding research is to reveal the genetic basis of phenotype, growth, production, and disease resistance traits, and apply the research results to genetic improvement. Most of the phenotype traits of fish are complex quantitative traits controlled by several genes. Therefore, the most important goal of catfish genetics research is to predict and reveal the genetic basis for controlling complex phenotypes through genomic research. For the moment, QTL mapping and GWAS research are two of the most important methods for correlating genetic and phenotypic variations [35]. Screening of SNPs or genes associated with phenotypes plays an important role in revealing the genetic basis and genetic application of phenotype traits [36].

In this study, the offspring of different families with similar initial body weights were cultured in the same culture environment for one year, and then the whole genome of 303 catfish was resequenced. The association between growth traits and SNPs in the whole genome was analyzed. Finally, 10 and 18 significant molecular markers were located on chromosome 20. The SNPs of the *rrp44* gene on chromosome 20 at 14658012 and 14657971 were significantly correlated with the two growth traits. The *rrp44* gene is an important rhythm regulation gene [37]. As a conserved exoribonuclease and exosome catalytic subunit, the *rrp44* gene is involved in the degradation and processing of RNA in nucleus and cytoplasm. The biological clock is mainly composed of RNA synthesis and degradation system. RNA degradation is the main method of gene feedback regulation in the biological clock. In Neurospora, *rrp44* is involved in the feedback regulation of the frq gene, which regulates the circadian rhythm of Neurospora according to the light cycle. There are important food-entrainable oscillators (FEOs) in animals. After the formation of an FEO, the animal can predict the time of food delivery, that is, food-anticipatory activity (FAA), which is the movement state of animals before food delivery. Eriksson observed that brown bullhead (*Ictalurus nebulosus*) were active in the early morning and dusk and could eat regularly even in a continuous dark environment [38]. Zebrafish possess an endogenous FEO; a strict feeding time can stimulate zebrafish to show FAA, and this rhythm can continue after the disappearance of food stimulation [39]. A similar phenomenon has been found in goldfish [40].

Animals with a strict feeding rhythm generally present strong FAA. In goldfish [39], zebrafish [40], mice [41], and other model organisms, it has been proved that animals with FAA have a strong desire for food and grow faster. Disruption of feeding rhythm leads to disordered feeding and slow growth rate. Based on this, it is speculated that the *rrp44* gene, which is significantly associated with growth traits, may play an important role in the regulation of feeding rhythm of channel catfish. Individuals with strict feeding rhythms have a faster growth rate. The results also suggest the importance of maintaining “timing, fixed-point and quantitative” feeding principles in the actual rearing process.

It is important to note that the association observed between *rrp44* SNPs and growth traits does not establish causation. While the link between *rrp44* and circadian/feeding rhythms is supported by studies in model organisms (e.g., *Neurospora*, zebrafish, goldfish), direct evidence in channel catfish is currently lacking. Therefore, the proposed role of *rrp44* in regulating feeding rhythms and growth in channel catfish remains a hypothesis that requires functional validation through future studies, including behavioral assays of food anticipatory activity (FAA), and potentially gene knockout or knockdown experiments.

## 5. Conclusions

This study identified 15 and 28 significant SNPs for monthly weight gain and body depth, respectively, with key loci on chromosome 20. Two SNPs in the *rrp44* gene were significantly associated with both traits, implicating it as a key growth regulator potentially via feeding rhythm control. These findings provide valuable molecular markers for genomic selection to accelerate genetic improvement in catfish aquaculture.

## Figures and Tables

**Table 1:** Raw and filter SNP number on each chromosome.

Chromosome	Raw SNP Number	Filter SNP Number	SNP Density (SNPs/KB)
chr1	611,468	293,696	8.91
chr2	169,763	73,698	6.52
chr3	430,368	187,596	7.39
chr4	261,213	120,831	7.15
chr5	401,440	194,408	8.46
chr6	423,631	199,473	8.78
chr7	462,703	215,969	8.47
chr8	418,761	194,696	8.05
chr9	571,735	293,636	9.32
chr10	517,762	242,187	7.27
chr11	340,339	178,393	9.53
chr12	390,009	192,356	8.44
chr13	664,128	309,930	7.58
chr14	599,024	289,242	7.94
chr15	371,459	174,259	9.30
chr16	130,133	55,825	7.72
chr17	521,979	234,140	7.79
chr18	544,882	253,720	7.76
chr19	461,702	233,697	8.19
chr20	438,606	198,960	8.67
chr21	129,476	56,982	3.94
chr22	547,709	259,647	7.92
chr23	260,768	124,622	7.37
chr24	544,685	246,321	7.37
chr25	319,702	145,703	8.35
chr26	413,709	178,626	7.60
chr27	145,105	61,310	7.27
chr28	394,012	173,305	7.64
chr29	559,588	258,483	8.86
Total/Even	12,045,859	5,641,711	8.01

Note: Chr.: Chromosome.

**Table 2:** Summary of the SNPs associated with monthly weight gain (MWG) and body depth (BH) in channel catfish G_2_ breeding population.

SNP Location	Chr.	Traits	REF	ALT	MAF	*p*-Value
14,658,012	20	MWG	C	G	0.20132	1.97 × 10^−9^
14,657,971	20	MWG	C	T	0.19970	1.45 × 10^−8^
7,569,563	20	MWG	A	T	0.05281	1.74 × 10^−7^
7,244,724	12	MWG	A	C	0.34160	2.31 × 10^−7^
324,296	18	MWG	C	A	0.22110	2.35 × 10^−7^
16,220,962	20	MWG	T	C	0.14520	2.44 × 10^−7^
20,326,756	20	MWG	C	T	0.09406	2.93 × 10^−7^
15,598,721	20	MWG	G	A	0.22440	3.88 × 10^−7^
160,322	22	MWG	T	A	0.11550	5.35 × 10^−7^
15,738,811	20	MWG	G	A	0.15350	6.21 × 10^−7^
64,155	1	MWG	A	C	0.32670	7.01 × 10^−7^
7,640,414	20	MWG	T	C	0.13200	7.35 × 10^−7^
15,894,102	20	MWG	A	T	0.23100	8.88 × 10^−7^
17,775,812	20	MWG	A	C	0.32510	9.12 × 10^−7^
143,510	22	MWG	C	T	0.33990	9.94 × 10^−7^
7,569,563	20	BH	A	T	0.05281	2.72 × 10^−9^
14,658,012	20	BH	C	G	0.20132	4.11 × 10^−9^
14,765,610	20	BH	G	T	0.14690	1.68 × 10^−8^
16,170,915	20	BH	C	T	0.22610	1.73 × 10^−8^
16,171,004	20	BH	T	G	0.23100	2.33 × 10^−8^
15,738,811	20	BH	G	A	0.15350	9.34 × 10^−8^
15,465,075	20	BH	C	T	0.09241	2.15 × 10^−7^
13,911,808	20	BH	G	A	0.05116	2.20 × 10^−7^
15,425,136	20	BH	C	T	0.09901	2.60 × 10^−7^
13,527,158	12	BH	A	T	0.20960	2.69 × 10^−7^
144,838	20	BH	G	A	0.06271	2.95 × 10^−7^
24,335,204	22	BH	G	A	0.08251	3.18 × 10^−7^
7,467,430	20	BH	T	C	0.06436	3.61 × 10^−7^
14,912,459	20	BH	T	G	0.06436	3.61 × 10^−7^
15,425,537	20	BH	G	A	0.10400	4.58 × 10^−7^
15,425,538	20	BH	G	T	0.10400	4.58 × 10^−7^
14,657,971	20	BH	C	T	0.19970	4.83 × 10^−7^
15,884,756	23	BH	G	A	0.10070	4.98 × 10^−7^
14,697,868	20	BH	G	A	0.10730	5.03 × 10^−7^
14,853,343	12	BH	T	G	0.11720	5.56 × 10^−7^
16,172,201	20	BH	C	T	0.31350	5.58 × 10^−7^
14,838,590	20	BH	T	C	0.09076	6.52 × 10^−7^
7,391,188	12	BH	C	T	0.2244	6.98 × 10^−7^
15,421,215	20	BH	T	C	0.2046	7.08 × 10^−7^
7,640,414	20	BH	T	C	0.1320	7.15 × 10^−7^
9,304,922	20	BH	C	T	0.0561	7.97 × 10^−7^
14,765,723	20	BH	T	C	0.1452	8.95 × 10^−7^
3,234,132	14	BH	C	T	0.1106	9.66 × 10^−7^

Note: REF: Reference allele; ALT: alternative allele; MAF: minor allele frequency.

## Data Availability

The raw sequencing data generated in this study have been deposited in the National Center for Biotechnology Information (NCBI) Sequence Read Archive (SRA) under BioProject accession number PRJNA1211191.
